# Comparison of the predictive performance of the BIG, TRISS, and PS09 score in an adult trauma population derived from multiple international trauma registries

**DOI:** 10.1186/cc12813

**Published:** 2013-07-11

**Authors:** Thomas Brockamp, Marc Maegele, Christine Gaarder, J Carel Goslings, Mitchell J Cohen, Rolf Lefering, Pieter Joosse, Paal A Naess, Nils O Skaga, Tahnee Groat, Simon Eaglestone, Matthew A Borgman, Philip C Spinella, Martin A Schreiber, Karim Brohi

**Affiliations:** 1 Department of Traumatology and Orthopedic Surgery, Cologne Merheim Medical Center (CMMC), University of Witten/ Herdecke, Ostmerheimer Str. 200, D-51109 Cologne, Germany; 2 Department of Traumatology, Division of Critical Care, Oslo University Hospital Ulleval, Kirkeveien 166, 0407 Oslo, Norway; 3 Department of Anesthesiology, Division of Emergency and Critical Care, Oslo University Hospital Ulleval, Kirkeveien 166, 0407 Oslo, Norway; 4 Trauma Unit, Department of Surgery, Academic Medical Center, University of Amsterdam, Meibergdreef 9, 1105 AZ Amsterdam, The Netherlands; 5 Department of Surgery, San Francisco General Hospital, University of California San Francisco (CA), Campus Box 0807, San Francisco CA 94143-0807, USA; 6 Department of Surgery Division of Trauma, Critical Care & Acute Care Surgery Oregon Health & Science University 3181 SW Sam Jackson Park Road, Mail Code L611 Portland, OR 97239, USA; 7 Department of Pediatrics, San Antonio Military Medical Center, 3551 Roger Brooke Drive, San Antonio, TX 78234, USA; 8 Associate Professor of Pediatrics, Director, Translational Research Program Division of Pediatric Critical Care Washington University in St Louis, St Louis Children's Hospital, St Louis, MO, USA 63110 United States Institute for Surgical Research, Fort Sam Houston, Texas, USA 78234; 9 Trauma Sciences, Bart's and the London School of Medicine and Dentistry, Queen Mary University of London, 4 Newark Street, London, E1 2AT, UK; 10 On behalf of the International Trauma Research Network (INTRN

## Abstract

**Background:**

The BIG score (Admission base deficit (B), International normalized ratio (I), and Glasgow Coma Scale (G)) has been shown to predict mortality on admission in pediatric trauma patients. The objective of this study was to assess its performance in predicting mortality in an adult trauma population, and to compare it with the existing Trauma and Injury Severity Score (TRISS) and probability of survival (PS09) score.

**Materials and methods:**

A retrospective analysis using data collected between 2005 and 2010 from seven trauma centers and registries in Europe and the United States of America was performed. We compared the BIG score with TRISS and PS09 scores in a population of blunt and penetrating trauma patients. We then assessed the discrimination ability of all scores via receiver operating characteristic (ROC) curves and compared the expected mortality rate (precision) of all scores with the observed mortality rate.

**Results:**

In total, 12,206 datasets were retrieved to validate the BIG score. The mean ISS was 15 ± 11, and the mean 30-day mortality rate was 4.8%. With an AUROC of 0.892 (95% confidence interval (CI): 0.879 to 0.906), the BIG score performed well in an adult population. TRISS had an area under ROC (AUROC) of 0.922 (0.913 to 0.932) and the PS09 score of 0.825 (0.915 to 0.934). On a penetrating-trauma population, the BIG score had an AUROC result of 0.920 (0.898 to 0.942) compared with the PS09 score (AUROC of 0.921; 0.902 to 0.939) and TRISS (0.929; 0.912 to 0.947).

**Conclusions:**

The BIG score is a good predictor of mortality in the adult trauma population. It performed well compared with TRISS and the PS09 score, although it has significantly less discriminative ability. In a penetrating-trauma population, the BIG score performed better than in a population with blunt trauma. The BIG score has the advantage of being available shortly after admission and may be used to predict clinical prognosis or as a research tool to risk stratify trauma patients into clinical trials.

## Background

The early prediction of mortality in trauma patients is challenging but has important potential benefits. The utility of existing mortality-prediction tools is confined to retrospective applications such as quality assessment, as they rely on variables not available in the early phases of care (such as the injury severity score). Accurate early prediction of the risk of death might have the potential to inform triage decisions, inform treatment, or stratify patients for further care. In particular, it would be attractive as an entry criterion for clinical trials to match an intervention to an appropriate at-risk population.

The BIG score (Admission base deficit (B), International normalized ratio (I), and Glasgow Coma Scale (G)) is a mortality-predicting score that has been shown to predict mortality accurately on admission in a cohort of pediatric trauma patients from a military trauma system. The BIG score performed better than other pediatric trauma scoring systems and was validated in a separate pediatric population with similar accuracy [1]. The BIG score has not been applied to adults, and its accuracy has not been compared with that of existing trauma mortality-prediction tools.

The first aim of this study was to assess whether the BIG score can predict mortality in an adult trauma population and to compare the predictive ability of the BIG score with the commonly used mortality-predicting Trauma and Injury Severity Score (TRISS; Trauma Score and Injury Severity Score (ISS) based on the ISS and the Revised Trauma Score (RTS), age and injury mechanism) and PS09 (Probability of Survival; model 09 based on ISS, GCS, age, gender and intubation) score [1-5].

A second aim was to evaluate and compare the ability to predict mortality of all scores on different subgroups.

## Materials and methods

### Data collection

A data-collection template was developed to collect all needed parameters from the participating sites. All primary admitted trauma-team activation patients aged 18 years or older during the period 2005 to 2010, inclusive, were eligible. Only patients with available and complete datasets for the calculation of the analyzed scoring systems (BIG, TRISS, and PS09) were included in the study (*n *= 15,730). In a second step, only patients with an ISS ≥4 were considered. This requires at least an AIS 2 type of injury and excludes all minor injuries. Finally, data from 12,206 patients from one military and six civilian trauma centers and registries in Europe and the United States were collected and retrospectively analyzed. We used 30-day mortality as the primary outcome parameter of our analysis. We then compared the BIG score against the TRISS and PS09 score on a representative population of trauma patients. Subgroup analysis only on patients with blunt or penetrating trauma was additionally done.

### Trauma centers and registries

Data were collected from four trauma centers participating in the International Trauma Research Network (INTRN; Amsterdam, Oslo, London, San Francisco), from the German TraumaRegister DGU (TR-DGU) and also from two participating registries in the United States (Joint Theater Trauma Registry (JTTR) and the Trauma Registry of the Oregon Health & Science University, Portland, OR). All participating sites are level-1 trauma centers.

### INTRN

The International Trauma Research Network (INTRN) is a formal academic network of high-volume trauma centers across Europe and the United States. The group was formed in 2009 and has grown strategically, developing fundamental, translational, and clinical research programs that span the complete breadth of trauma disciplines [6,7].

### TraumaRegister DGU

The TraumaRegister DGU (TR-DGU) is a prospective multicenter database with standardized documentation of patients with severe trauma and thus requiring intensive care. This registry comprises detailed information on demographics and clinical and laboratory data. Data from the TraumaRegister DGU include patients from about 108 trauma units around Germany [8].

### OHSU Trauma Registry

The Oregon Health and Science University (OHSU) Trauma Registry contains information from more than 45,000 patients treated since 1985. The registry contains detailed information for each patient concerning prehospital, ED, and in-hospital care. All research projects are approved by an Institutional Review Board (IRB).

### JTTR

The Joint Theatre Trauma Registry (JTTR) was established by the Department of Defense to collect comprehensive data on all personnel, military and civilian, admitted to military treatment facilities within Iraq and Afghanistan. It is maintained at the US Army Institute of Surgical Research in San Antonio, Texas, USA.

Data are handled anonymously, and case identification is possible only through the participating hospital.

### Trauma scores

For our analysis, we compared the original pediatric BIG score with the commonly used TRISS and PS09 scores [1,2,9]. In a second approach, we tested the mortality prediction of the BIG score on our blunt-trauma and separately on our penetrating-trauma patients and compared the score again with the TRISS and the PS09 scores (Table 1).

**Table 1:** Variables of all scores.

BIG Score	TRISS	PS09 Score
BE	ISS	ISS
INR	Age	Age
GCS	Injury mechanism	GCS
	RTS	Gender
		Intubation

BE: Base Excess; INR: International Normalized Ratio; GCS: Glasgow Coma Scale; ISS: Injury Severity Score; RTS: Revised Trauma Score.

### The BIG score

The pediatric BIG score is a mortality-predicting score for children with traumatic injuries. It was developed by Borgman and colleagues in 2011. They retrospectively analyzed data from 2002 to 2009 and found that admission base deficit (B), international normalized ratio (I), and Glasgow Coma Scale (G) were independently associated with mortality. The variables were combined into the pediatric BIG score (base deficit + (2.5 × international normalized ratio) + (15 Glasgow Coma Scale)). This equation can then be implemented into a mortality-predicting formula: predicted mortality = 1/(1 + e^-x^), where *x *= 0.2 × (BIG score) - 5.208. A BIG score of <12 points suggests a mortality of <5%, whereas a cut-off of >26 points corresponds to a mortality of >50%. The BIG score can be performed rapidly on admission to evaluate severity of illness and to predict mortality in children [1].

### The TRISS method

Physiological and anatomic data are included in the Trauma Injury Severity Score (TRISS) that was published in 1987 by Boyd and colleagues [2] based on a North American population. Since then, the estimation of prognosis is also discussed critically, which led to several reevaluations of the score [10,11]. TRISS combines the variables anatomic injury (ISS), physiological derangement (RTS), patient age, and injury mechanism to predict survival from trauma [2,12]. TRISS quickly became the standard method for outcome assessment [12,13]. For our analysis, we used the TRISS method with its coefficients published by Champion and colleagues in 1990 (MTOS 1990) [14].

### The PS09 Score (Probability of Survival, Model: 09)

In 2006, Bouamra and colleagues [19] published a new survival prediction model based on data from the UK Trauma Audit and Research Network (TARN). Since 1989, TARN used TRISS as a score to predict outcome, initially with the MTOS 1990 coefficients, and later with UK TARN-derived TRISS coefficients. However, with the TRISS method, a large amount of data was lost. In the PS09 model, the prediction-model coefficients have been revised on recent data; the model still includes all those subsets by using age, a transformation of ISS, GCS, gender, and Gender × Age interaction as predictors [9].

### Statistical analysis

The statistical analysis of this study is based on the data from six civilian and one military database, extracting data from specified periods between 2005 and 2010. Demographic data are presented as means with standard deviation (SD) for continuous variables and as percentages for incidence rates. The *U *test was used for continuous variables, and the $\chi_{2}$ test for categoric variables. Statistical significance was set at *P *values less than 0.05. The quality of all scoring systems in predicting mortality was analyzed and presented in terms of discrimination and precision. Discrimination measures the ability of a scoring system to separate survivors from nonsurvivors. This was measured with the area under the receiver operating characteristic curves (AUROCs). The ROC curve summarizes the trade-off between sensitivity and specificity of a predictive score by using all score values as potential cut-off values. Its value varies between 0.5 (no discrimination) and 1.0 (perfect discrimination). AUROCs are presented with 95% CI, and differences between AUROC curves were evaluated by using a method derived by Hanley and colleagues [15]. In addition, the precision describes how well a score-based prognosis is able to meet the observed mortality rate. All statistical analyses were performed by using IBM SPSS 20 (IBM SPSS Inc, Chicago, IL, USA).

## Results

In total, 12,206 patients were included in the study. Of those, 4,949 patients were included by civilian trauma centers, and 7,257 (59%) patients were included by military trauma centers (Table 2).

**Table 2:** Characteristics of all databases.

Database	1	2	3	4	5	6	7
	Amsterdam	London	Oregon	Oslo	San Francisco	TR-DGU	JTTR
Site	Academic Medical Center Amsterdam, Netherlands	Royal London Hospital London, United Kingdom	Health & Science University Oregon, Portland US	University Hospital Ulleval Oslo, Norway	General Hospital San Francisco, California US	TraumaRegister DGU^® ^Germany	US Army Institute of Surgical Research, San Antonio, Texas US
Year of data collection	2005-2010	2005-2010	2008-2010	2008-2010	2005-2010	2005-2010	2005-2010
Civilian or military data	Civilian	Civilian	Civilian	Civilian	Civilian	Civilian	Military
Number of patients	609	1,483	142	544	247	1,924	7257

All seven databases are shown together with the number of patient data contributed by each site. n, total number of patients.

### Demographic data

Table 3 provides an overview of demographic and baseline physiological data of each database. Data are presented as means or as percentages. The mean ISS was 15 ± 11 with a 30-day mortality rate of 4.8%. Table 4 presents all variables associated with mortality. In total, 588 (4.8%) patients died. Survivors were younger (33.2 versus 50.4 years) and were more likely to sustain penetrating trauma (47.5% versus 26.0%). Nonsurvivors had a significantly higher ISS (33 versus 14; *P *< 0.001), a worse mean base excess (-6.9 versus -1.9; *P *< 0.001), and a lower GCS (7 versus 14; *P *< 0.001).

**Table 3:** Demographic data of all sites compared.

Variable		Total (*N *= 12,206)	Amsterdam (*n *= 609)	London (*n *= 1,483)	Oregon (*n *= 142)	Oslo (*n *= 544)	San Francisco (*n *= 247)	TRDGU (*n *= 1,924)	JTTR (*n *= 7,257)
Age (years)	Mean ± SD	34 ± 16	44 ± 18	39 ± 17	52 ± 18	44 ± 18	42 ± 19	46 ± 19	28 ± 11
Male	n (%)	10,784 (88.3)	432 (70.9)	1201 (63.8)	108 (76.1)	405 (74.4)	202 (81.8)	1432 (74.4)	7004 (96.5)
Penetrating trauma	n (%)	5666 (46.4)	41 (6.7)	216 (14.6)	15 (10.6)	36 (6.6)	81 (32.8)	98 (5.1)	5179 (71.4)
Intubated (on scene)	n (%)	1688 (13.9)	72 (11.8)	NA	NA	86 (15.8)	232 (93.9)	1057 (55.0)	232 (3.2)
Systolic Blood Pressure (mmHg)	Mean ± SD	128 ± 25	137 ± 27	129 ± 29	138 ± 32	133 ± 29	127 ± 39	124 ± 30	128 ± 21
Heart rate (bpm)	Mean ± SD	93 ± 22	87 ± 19	91 ± 25	92 ± 24	89 ± 19	98 ± 28	89 ± 21	94 ± 22
Respiratory rate (pm)	Mean ± SD	19 ± 7	17 ± 5	19 ± 7	19 ± 6	19 ± 7	21 ± 6	15 ± 6	20 ± 6
INR	Mean ± SD	1.2 ± 0.5	1.0 ± 0.4	1.1 ± 0.3	1.3 ± 0.5	1.1 ± 0.3	1.2 ± 0.4	1.2 ± 0.8	1.2 ± 0.4
Base excess (mmol/l)	Mean ± SD	2.2 ± 4.0	2.2 ± 3.7	2.2 ± 4.4	2.7 ± 3.5	-2.1 ± 3.1	6.6 ± 5.2	3.2 ± 4.3	-1.7 ± 3.8
Glasgow Coma Scale (total)	Mean ± SD	13 ± 3	13 ± 4	13 ± 4	13 ± 3	14 ± 3	9 ± 5	11 ± 5	14 ± 3
Injury Severity Score (ISS)	Mean ± SD	15 ± 11	15 ± 11	18 ± 11	24 ± 12	16 ± 11	28 ± 15	24 ± 14	12 ± 9
Lengh of stay on ICU (days)	Mean ± SD	6.2 ± 10.1	1.6 ± 4.1	2.9 ± 6.7	8.3 ± 9.3	3.8 ± 5.9	9.1 ± 8.9	10.4 ± 12.7	NA
Lengh of stay in hospital (days)	Mean ± SD	17.8 ± 22	9.9 ± 15.4	18.1 ± 25.3	16.1 ± 15.7	6.6 ± 8.1	21.1 ± 27.1	22.9 ± 20.8	NA
Mortality within 30 days	n (%)	588 (4.8)	30 (4.9)	80 (5.4)	9 (6.3)	22 (3.0)	71 (28.7)	242 (12.6)	139 (1.9)

Data are presented as mean and standard deviation (SD) or as percentage. ISS, Injury Severity Score; ICU, Intensive Care Unit; TRDGU, TraumaRegister DGU^® ^of the German Trauma Society; JTTR, Joint Theater Trauma Registry; INR, International Normalized Ratio.

**Table 4:** Variables associated with mortality.

Variable		Survivor (*n *= 11618)	Non-survivor (*n *= 588)	p-value
Age	Mean ± SD	33.2 ± 15.2	50.4 ± 22.9	< 0.001
Male	%	89.1	74.3	< 0.001
Penetrating trauma	%	47.5	26.0	< 0.001
Intubated (on scene)	%	11.9	55.8	< 0.001
Systolic Blood Pressure (mmHg)	Mean ± SD	129 ± 22	96 ± 35	< 0.001
Heart rate (bpm)	Mean ± SD	93 ± 22	96 ± 35	0.032
Respiratory rate (pm)	Mean ± SD	19 ± 6	16 ± 10	< 0.001
INR	Mean ± SD	1.1 ± 0.3	1.7 ± 1.6	< 0.001
Base excess mmol/l	Mean ± SD	1.9 ± 3.7	6.9 ± 6.5	< 0.001
Glasgow Coma Scale (total)	Mean ± SD	14 ± 3	7 ± 5	< 0.001
Injury Severity Score	Mean ± SD	14 ± 10	33 ± 16	< 0.001
Lengh of stay on ICU (days)	Mean ± SD	6.4 ± 10.4	4.5 ± 5.4	< 0.001
Lengh of stay in hospital (days)	Mean ± SD	19.1 ± 22.2	5.7 ± 11	< 0.001

Data are presented as mean or as percentage. ICU, Intensive Care Unit; INR, International Normalized Ratio.

### Quality criteria

Table 5 characterizes quality criteria for all scores compared. The table presents the discrimination and precision ability of the BIG, TRISS, and PS09 scores for all patients as well as for a blunt- and penetrating-trauma population. The AUROCof the BIG score and the TRISS score are significantly different (*P *< 0.001). The difference between the TRISS and PS09 scores is not statistically significant (*P *= 0.32) in the combined dataset.

**Table 5:** Quality criteria for all scores compared.

	BIG score	TRISS	PS09 score
**Discrimination**			

AUROC curve (95% CI) All patients (*n *= 12206)	0.892 (0.879 - 0.906)	0.922 (0.913 - 0.932)	0.925 (0.915 - 0.934)
AUROC curve (95% CI) Blunt trauma (*n *= 6540)	0.876 (0.859 - 0.892)	0.917 (0.906 - 0.928)	0.921 (0.911 - 0.932)
AUROC curve (95% CI) Penetrating trauma (*n *= 5666)	0.920 (0.898 - 0.942)	0.929 (0.912 - 0.947)	0.921 (0.902 - 0.939)

**Precision**			

All patients			
Predicted Mortality (%)	4.8	6.6	7.9
	
Observed Mortality (%; 95% CI)	4.8 (4.4-5.2)		
Blunt Trauma			
Predicted Mortality (%)	5.5	9.6	11.9
	
Observed Mortality (%; 95% CI)	6.7 (6.1-7.3)		
Penetrating Trauma			
Predicted Mortality (%)	4.0	3.2	3.4
	
Observed Mortality (%; 95% CI)	2.7 (2.3-3.1)		

Quality criteria for all scores on all data (*n *= 12,206), on all blunt (*n *= 6,540) and penetrating (*n *= 5,666) trauma patients. Data are presented as AUROC (Area under receiver operating characteristic curve) together with a 95% Confidence Interval (CI).

The expected mortality rate (precision) of the BIG score, PS09 score, and TRISS was compared with the observed mortality rate. In Table 5, the observed mortality of all patients is set by 4.8%. The expected mortality calculated for the BIG score is 4.8%, whereas the expected mortality of the TRISS and PS09 scores are 6.6% and 7.9%, respectively.

### Blunt trauma

Comparing only patients who sustained blunt trauma (*n *= 6,540), our analysis shows that the overall accuracy of the PS09 score had an AUROC of 0.921 (95% CI, 0.911 to 0.932), the TRISS score of 0.917 (95% CI, 0.906 to 0.928), and the BIG score had an AUROC of 0.876 (95% CI, 0.859 to 0.892). The difference between AUROC of the BIG score and the TRISS score is significant (*P *< 0.001). The difference between the TRISS and PS09 score is not statistically significant (*P *= 0.24) in the blunt dataset.

### Penetrating trauma

In patients with penetrating trauma (*n *= 5,666), the BIG score (0.920; 95% CI, 0.898 to 0.942) performed comparably to the PS09 score (0.921; 95% CI, 0.902 to 0.939). The TRISS score had an AUROC of 0.929 (95% CI, 0.912 to 0.947). The difference between AUROC results in the penetrating group was not significant (all *P *> 0.16).

### Military and civilian data

On a military dataset (*n *= 7,257), the BIG score had an AUROC of 0.929 (95% CI, 0.909 to 0.949), and the PS09 score had an AUROC of 0.922 (95% CI, 0.904 to 0.940) and TRISS of 0.915 (95% CI, 0.891 to 0.939) (all *P *> 0.31). On a civilian dataset (*n *= 4949), the PS09 score had a similar AUROC (0.901; 95% CI, 0.887 to 0.914) to the TRISS (0.896; 95% CI, 0.882 to 0.909; *P *= 0.24). The AUROC of the BIG score (0.849; 95% CI, 0.830 to 0.868) was significantly lower (*P *< 0.001) (Table 6).

**Table 6:** Military data vs.

	BIG score	TRISS	PS09 score
**Discrimination**			

AUROC curve (95% CI) Military data (*n *= 7257)	0.929 (0.909 - 0.949)	0.915 (0.891 - 0.939)	0.922 (0.904 - 0.940)
AUROC curve (95% CI) Civilian data (*n *= 4949)	0.849 (0.830 - 0.868)	0.896 (0.882 - 0.909)	0.901 (0.887 - 0.914)

Civilian data. AUROC results of all compared scores on all military and on all civilian data.

**Figure 1:**
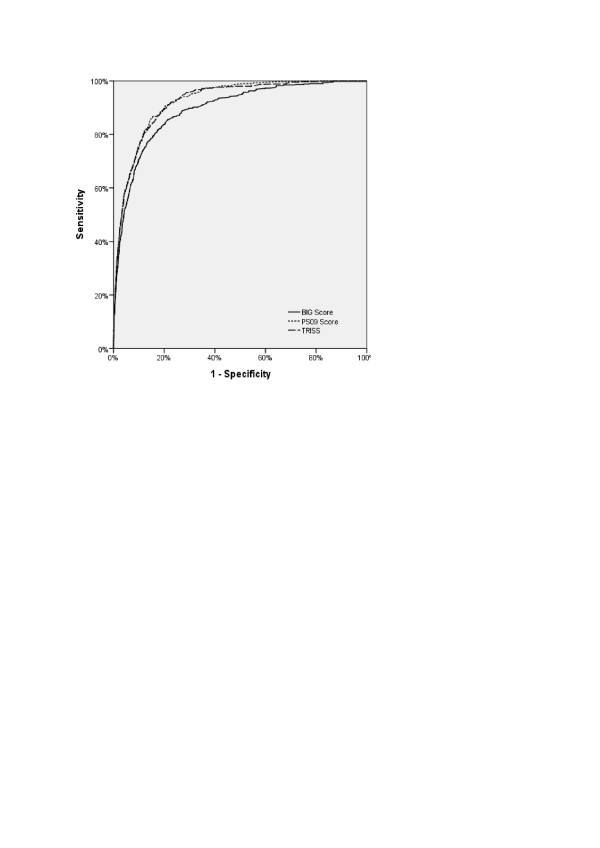
**Receiver operating characteristic (ROC) curves for the BIG, TRISS and PS09 score**.

Figure 1 depicts the AUROCs for the BIG, TRISS, and PS09 scores on all blunt and penetrating trauma patients combined.

## Discussion

For the first time, the performance of the BIG score was analyzed on an adult trauma population. Data from civilian and military trauma centers and registries with a representative trauma population of blunt and penetrating trauma were used for the current analysis. When all scores were tested on the whole dataset, the BIG score performed well in predicting mortality in the adult trauma population. Unlike complex scoring systems, the BIG score can be used directly on admission of a trauma patient, because it uses variables that are rapidly available for assessing severity of illness and predicting mortality. Time-consuming parameters like the ISS are not included within the BIG score. Hence, the BIG score might be used for trauma trials in which mortality is the intended primary outcome parameter.

In the present analysis, the BIG score was shown to perform well in predicting mortality in the penetrating-trauma population versus the blunt-trauma population. The TRISS and the PS09 scores slightly overpredicted mortality in all subgroups. The BIG score overpredicted mortality only in the penetrating-trauma group. In addition, the mortality prediction of the BIG score was more accurate on military trauma data than on civilian data. This might be due to its composition, as the BIG score includes parameters highly reflecting the two major causes of acute death from trauma (for example, brain injury and uncontrolled hemorrhage [1,16-18]). In assessing the epidemiology of trauma death, Sauaia and co-workers [19] identified central nervous system (CNS) injuries to represent the most frequent cause of death (42%), followed by exsanguination (39%). The BIG score reflects these observations by including INR and GCS in the calculation. The INR provides information about the hemocoagulative status of the patient [17,20,21], whereas the GCS is used as a surrogate marker for the level of consciousness and to estimate its severity, due to either traumatic brain injury (TBI) or severe hypoperfusion [22-24]. However, the GCS was recently challenged, and the role as a tool to reflect the mental status of a trauma patient is widely discussed [25].

The third parameter of the BIG score, base deficit (BD), was shown to be a valuable indicator of shock, abdominal injury, fluid requirements, efficacy of resuscitation, and a predictor of mortality after trauma [26-31].

In the past, several scoring systems and algorithms have been developed to predict mortality in the trauma population. The one most closely related to the BIG score is the Emergency Trauma Score (EMTRAS), developed by Raum and colleagues [32] from data derived from the TraumaRegister DGU. This score includes similar components to the BIG score, including GCS, base excess (BE), and prothrombin time (PT), as well as age. When this score was compared with the Revised Trauma Score (RTS), ISS, NISS, and TRISS scores with regard to mortality after trauma, the EMTRAS was superior. However, the EMTRAS was developed and validated on one single and retrospective database and therefore has not been validated externally or prospectively [32]. Perel and co-workers [33] recently developed a prognostic model for early death in patients with traumatic bleeding on a dataset from the Clinical Randomisation of an Antifibrinolytic in Significant Haemorrhage (CRASH-2) trial and validated the score on 14,220 selected trauma patients from the Trauma Audit and Research Network (TARN). Glasgow coma scale score, age, and systolic blood pressure were the strongest predictors of mortality. A chart was constructed to provide the probability of death at the point-of-care. Future research must evaluate whether the use of this prognostic model in clinical practice has an effect on the management and outcomes of trauma patients [33].

Most other scoring systems and algorithms to predict mortality in the trauma population on admission are limited because of their complexity and the high number of variables included for calculation. With this, the Trauma Injury Severity Score (TRISS), for example, offers a standard approach for evaluating outcome of trauma care [2]. Similar to the TRISS, the PS09 score uses a combination of anatomic and physiological parameters. However, information on the entire and complete injury pattern is usually difficult to obtain in the acute phase of Emergency Department (ED) care and requires potentially time-consuming imaging technology. Limitations of both scores are multiple and widely discussed in the literature [2,9,10]. Of note, it also takes trained personnel significant time to review charts and calculate complex scores, so that the clinical use of these approaches has to be questioned.

Limitations of the present study are the same that are inherent in retrospective reviews using registry data. To generate the dataset for the present analysis, a number of patients had to be excluded as a result of missing data in the contributing registries, resulting in a selection bias of patients. Only patients with complete datasets were included in the study. In a subgroup analysis, we excluded patients with minor injuries (ISS < 4), which reduced the dataset by about 20%. These excluded patients are most likely survivors. Trauma mortality scores must be evaluated on a trauma population with a certain amount of injury severity to predict mortality accurately. On a trauma population without an ISS limitation, we proved that all three scores had slightly better AUROC results, because outcome prediction is easy in the group of patients with minor injuries.

The fact that one registry (San Francisco) obviously contributed more severely injured patients with impaired outcome into the joint dataset might also have biased the results. A similar effect may have been related to the contribution of more penetrating-trauma patients derived from the military database.

The BIG score waives the need for anatomic classifications and physiologic parameters, like systolic blood pressure, heart rate, or respiratory rate. The GCS is a simple clinical assessment, whereas BE and INR can easily and quickly be obtained from point-of-care devices in the ED setting. These results are usually known within minutes of ED arrival. The BIG score does not require any variables that are not readily available in the acute phase of injury care (for example, ISS, NISS). Therefore, we think that the BIG score can be used to identify trauma patients at risk. However, the BIG score was shown to predict mortality accurately in an adult trauma population, and it may be used to determine inclusion criteria for prospective acute care research studies.

## Conclusions

Our results show that the BIG score, initially developed and validated in the pediatric trauma population, can also predict mortality in the adult trauma population. The BIG score performs well compared with complex scoring systems like the TRISS and PS09 scores, although it has significantly less discriminative ability. The score was shown to perform superiorly in the penetrating trauma-population and on a military dataset, which could make it a useful system in combat casualties, for whom time and resources are limited. In addition, the BIG score can be used independent of injury severity, and it can be used to determine inclusion criteria for prospective acute care research studies.

## Key messages

• This study validated three mortality-predicting scores on a multicenter database with military and civilian data.

• The BIG score has been shown to predict mortality in pediatric trauma patients. Our analysis show that this score can also be used on a representative adult trauma population.

• In the present analysis, the BIG score was shown to perform well in predicting mortality in the penetrating trauma population versus the blunt trauma population.

• The BIG score is based on information available shortly after admission.

• Mortality predicting scores that are quickly available in the Emergency Department can be a useful tool to include patients in acute care research studies.

## Abbreviations

AIS: Abbreviated Injury Scale; AUROC: area under the receiver operating characteristic; BD: base deficit; BE: base excess; BIG: base excess: International normalized ratio: Glasgow coma scale; CI: confidence interval; CNS: central nervous system; ED: emergency department; EMTRAS: Emergency Trauma Score; GCS: Glasgow Coma Scale; ICU: intensive care unit; INR: International Normalized Ratio; INTRN: International Trauma Research Network; ISS: Injury Severity Score; JTTR: Joint Theatre Trauma Registry; NISS: New Injury Severity Score; OHSU: Oregon Health & Science University; PS: probability of survival; PT: prothrombin time; ROC: receiver operating characteristic; RTS: Revised Trauma Score; TARN: Trauma Audit and Research Network; tbi: traumatic brain injury; TR-DGU: TraumaRegister DGU of the German Trauma Society (DGU).

## Competing interests

The authors declare that they have no competing interests.

## Authors' contributions

TB contributed to study design, acquisition and interpretation of data, recording of paper and analyzing data. PS, KB, MM, MS, and MB conceived of the study, provided statistical advice on study design, and analyzed data. RL, KB, and MM contributed to analysis and interpretation of data and revision of the article. All other authors contributed to data collection and to manuscript revision. All authors read and approved the final manuscript for publication.
